# Non‐Equilibrium Synthesis of Whitlockite Assisted by Localized H_2_O Vapor Pressure

**DOI:** 10.1002/advs.76175

**Published:** 2026-06-16

**Authors:** Min‐Jung Kim, Minwoo Lee, In‐Ho Jung, Seung‐Kyun Kang, Ji‐Soo Jang, Hyung‐Seop Han

**Affiliations:** ^1^ Biomaterials Research Center Biomedical Research Division Korea Institute of Science and Technology (KIST) Seoul Republic of Korea; ^2^ Department of Materials Science and Engineering College of Engineering Seoul National University Seoul Republic of Korea; ^3^ Electronic and Hybrid Materials Research Center Korea Institute of Science and Technology (KIST) Seoul Republic of Korea; ^4^ SKKU Advanced Instituted of Nanotechnology and Department of Nanoengineering Sungkyunkwan University Suwon Republic of Korea

**Keywords:** calcium phosphates, IPL, photothermal shock synthesis, whitlockite

## Abstract

Whitlockite (Ca_18_Mg_2_(HPO_4_)_2_(PO_4_)_12_) has attracted considerable attention in the fields of bone grafting and bone regeneration due to the presence of magnesium ions and the HPO_4_^2−^ group. However, conventional methods for synthesizing whitlockite require strict conditions and often result in excessive particle growth and compositional inhomogeneity. Here, we report the ultrafast and precisely controlled synthesis of whitlockite using a photothermal shock process induced by intense pulsed light (IPL). The formation of the whitlockite phase indicates that the precursor particles underwent a transient liquid phase and ion diffusion during IPL irradiation, facilitating the emergence of the new phase. Furthermore, we demonstrate that the H_2_O‐rich, high‐pressure environment generated at the interface of carbon microheaters and the hydrated precursor plays a key role in whitlockite formation. This simple and rapid IPL‐assisted approach provides an effective strategy for calcium phosphate crystallization and bioceramic fabrication, opening new avenues for controlled synthesis of complex phosphate phases.

## Graphical abstract

A carbon substrate acts as a microheater during intense pulsed light irradiation, enabling rapid heating and cooling for whitlockite formation. In contrast to conventional white ceramic synthesis with weak photothermal response, this platform concentrates thermal energy locally and promotes phase‐selective crystallization under ultrafast, nonequilibrium conditions.

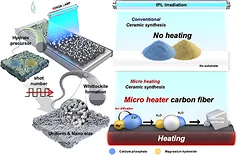

**Abbreviations**
β–TCP: β–tricalcium phosphate
DCPA: Dicalcium phosphate anhydrous
DCPD: Dicalcium phosphate dihydrate
FE–SEM: Field Emission Scanning Electron Microscope
GIXRD: Grazing Incidence X‐ray diffraction
HA: Hydroxyapatite
IPL: Intense pulsed light
TCP: Tricalcium phosphate
TEM: Transmission Electron Microscope
TGA: Thermogravimetric Analysis
XPS: X‐ray Photoelectron Spectroscopy
XRD: X‐ray diffraction

## 1 Introduction

Whitlockite (Ca_18_Mg_2_(HPO_4_)_2_(PO_4_)_12_) is the second most abundant calcium phosphate phase found in human bone, and has attracted considerable attention due to its excellent biocompatibility and superior osteogenic potential [1, 2, 3]. A distinctive feature of whitlockite is the presence of magnesium ions, which are known to enhance angiogenesis during the early stages of bone regeneration [4]. In addition, the HPO_4_^2−^ groups—absent in other calcium phosphates such as hydroxyapatite (HA) or β‐tricalcium phosphate (β–TCP)—facilitate faster ion release, supplying Ca^2+^ and PO_4_^3−^ ions crucial for new bone mineralization [5, 6]. Owing to these unique chemical features, whitlockite is regarded as a promising ceramic phase for diverse biomedical applications, particularly in bone grafting and bone regeneration [7].

Despite its favorable biological characteristics, practical utilization of whitlockite remains challenging because of the complexity of its synthesis [8]. The most established approach is wet‐chemical precipitation, which demands precise control over parameters such as precursor concentration, pH, temperature, and aging time [9]. Kim et al. reported that successful whitlockite formation requires a temperature of approximately 90°C, pH 4–5, carefully adjusted precursor ratios, and an aging step of roughly 20 h [1]. Furthermore, an additional annealing step is typically required to enhance crystallinity [10]. Even under these stringent conditions, the overall process typically requires several days to a week and may still yield undesired byproducts such as HA or β–TCP [11]. Thus, the extreme sensitivity of the reaction system and stringent control requirements make it difficult to achieve reproducibility and consistency.

In another case, solid‐state synthesis methods based solely on thermal treatment have been investigated as alternative approaches [12]. These methods yield particles with a broad particle size distribution (on the order of tens of micrometers), and the formation mechanism of the solid‐solution phase of whitlockite has not yet been clearly elucidated [13]. These limitations are critical because nanosized calcium phosphate, including whitlockite, closely resembles extracellular matrices and natural bone crystals, thereby providing a highly biocompatible microenvironment [14]. Its high surface‐area‐to‐volume ratio enables rapid ion release, which in turn promotes bone regeneration [15]. Therefore, the development of a faster, more straightforward, and better‐controlled synthesis method remains essential for advancing nanosized whitlockite toward practical biomedical applications.

Recently, several studies have explored non‐equilibrium synthesis induced like pulsed laser, intense pulsed light, as a powerful platform for the rapid formation of functional nanomaterials far from equilibrium conditions [16, 17, 18]. This technique employs a xenon flash lamp to deliver high‐energy light pulses (≤20 ms), enabling simultaneous rapid heating and ultrafast cooling of the material [19]. In general, IPL has been primarily used for synthesizing metal‐based nanoparticles [20, 21], with well‐established applications in catalysis [22, 23], energy storage [24, 25], and ink jet processes, where its effectiveness as a rapid sintering tool has been well demonstrated [26, 27, 28]. In contrast, whitlockite and other calcium phosphate ceramics, as typical wide bandgap materials, present a fundamental challenge because of their poor light absorption [29]. As a result, photothermal synthesis or sintering of such materials becomes considerably more challenging. Nevertheless, when appropriately optimized, IPL could offer a unique and ultrafast alternative to conventional ceramic synthesis methods. It is also well established that the HPO_4_^2−^ groups in the calcium phosphate structures are stabilized under conditions of high water activity [30, 31]. Therefore, IPL offers the potential to achieve nanoscale particle formation within mere milliseconds, representing a transformative advance toward efficient bio‐ceramic synthesis.

In this work, we present a simple and powerful strategy for the rapid synthesis of whitlockite by creating a local high‐H_2_O‐vapor‐pressure environment. To overcome the intrinsically poor photothermal response of calcium phosphate‐based ceramics, we employed a carbon substrate that acts as a microheater, efficiently absorbing flash pulses and rapidly transferring the resulting thermal shock to the precursor. This process induces rapid partial melting and facilitates the formation of a new crystalline phase. More importantly, the combination of hydrated precursors with IPL irradiation generates a transient, highly H_2_O‐rich environment, which plays a key role in providing a favorable environment for whitlockite formation. This approach not only overcomes the challenges associated with the conventional synthesis methods but also provides a pathway toward nanoscale and fine‐structured whitlockite as a next‐generation biomaterial.

## 2 Results and Discussion

### 2.1 IPL‐Driven Whitlockite Synthesis Enabled by a Carbon Microheater

We synthesized whitlockite using the photothermal method through IPL. Hydrated calcium phosphate dihydrate (DCPD, CaHPO_4_·2H_2_O) and magnesium hydroxide (Mg(OH)_2_) served as precursors. Carbon fiber acted as an active microheater that converted light into heat, enabling instantaneous temperature rise (Scheme 1a). As shown in Scheme 1b, precursors without substrate exhibit low optical absorption, and their direct photothermal response is poor. Thus, these two precursors themselves cannot absorb light and therefore cannot generate appropriate heat for phase formation. To overcome this, efficient photothermal carbon fiber paper was used as the substrate. Upon irradiation, the carbon rapidly converted the IPL pulses to heat and delivered it across the near interface. This process transiently partially melts the contacting precursors and promotes ion diffusion, wherein Mg^2+^ ions substitute Ca^2+^ ion sites, ultimately leading to the crystallization of whitlockite (Scheme 1c). This microheater‐assisted synthesis allows ultrafast, millisecond‐scale formation, particularly when the precursor and substrate are properly optimized.

**SCHEME 1:**
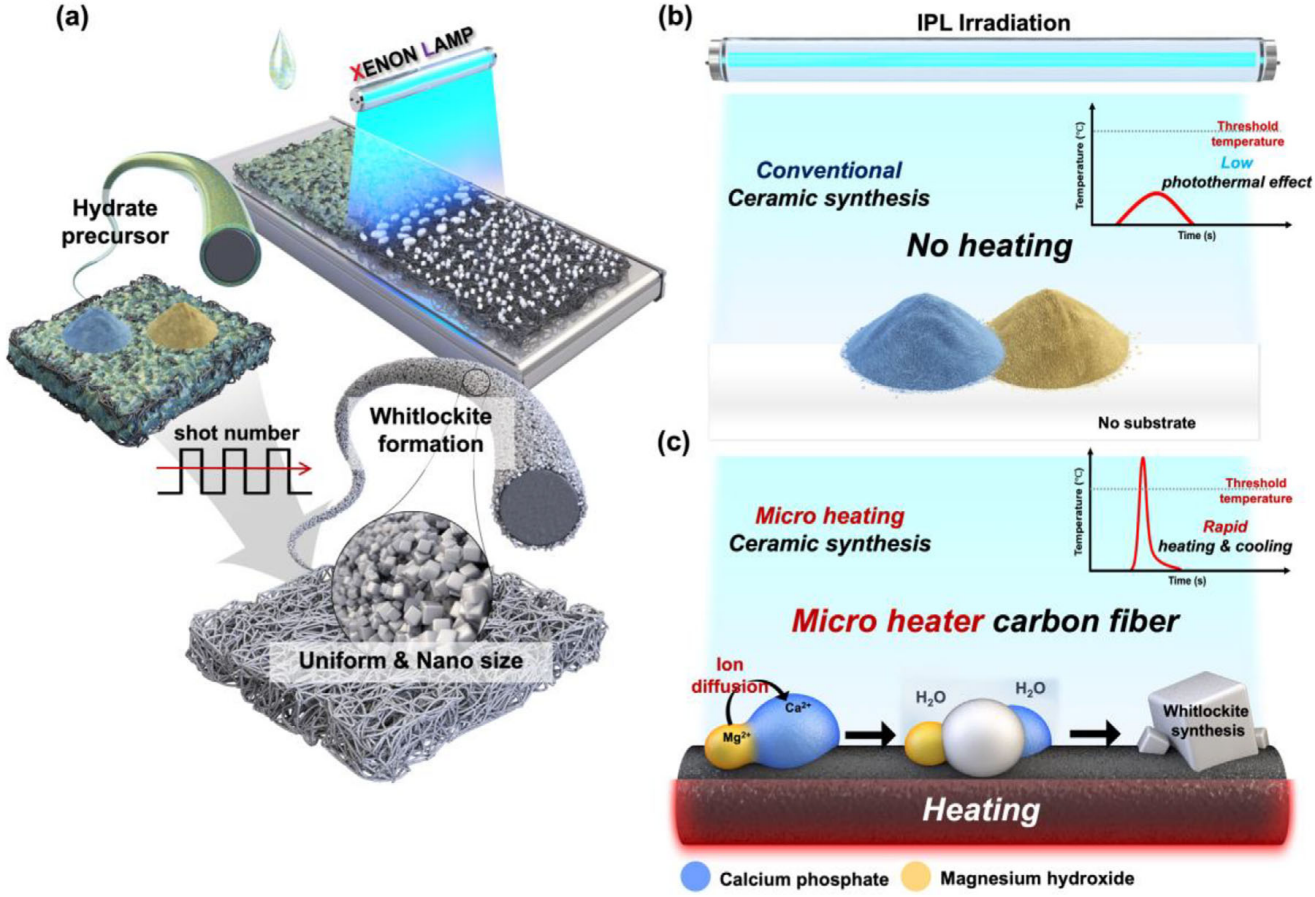
IPL‐driven whitlockite synthesis enabled by a carbon microheater. (a) Schematic illustration of the whitlockite synthesis process using IPL. (b) In conventional white ceramic synthesis, heat generation is insufficient, resulting in poor photothermal effects. (c) By synthesizing ceramics on a carbon fiber substrate, micro‐heating enables a rapid heating and cooling process, leading to the formation of the whitlockite phase.

### 2.2 Optimization of IPL Conditions for Whitlockite Synthesis

To prepare whitlockite synthesis via IPL, we used the following process, as shown in Figure 1a. First, CaHPO_4_·2H_2_O and Mg(OH)_2_ were placed in a mortar and pestle and mixed vigorously with ethanol. The mixed powders were dispersed at a concentration of 10 mg mL^−1^ in ethanol and dropped three times (100 µL each) onto carbon paper prepared in a 1 × 2 cm square. The samples were then dried at 63.5°C for 1 h. Subsequently, the prepared sample was placed between glasses and exposed to a xenon flash lamp under the desired IPL conditions. More details of sample preparation can be found in the **Experiment Section**.

**FIGURE 1:**
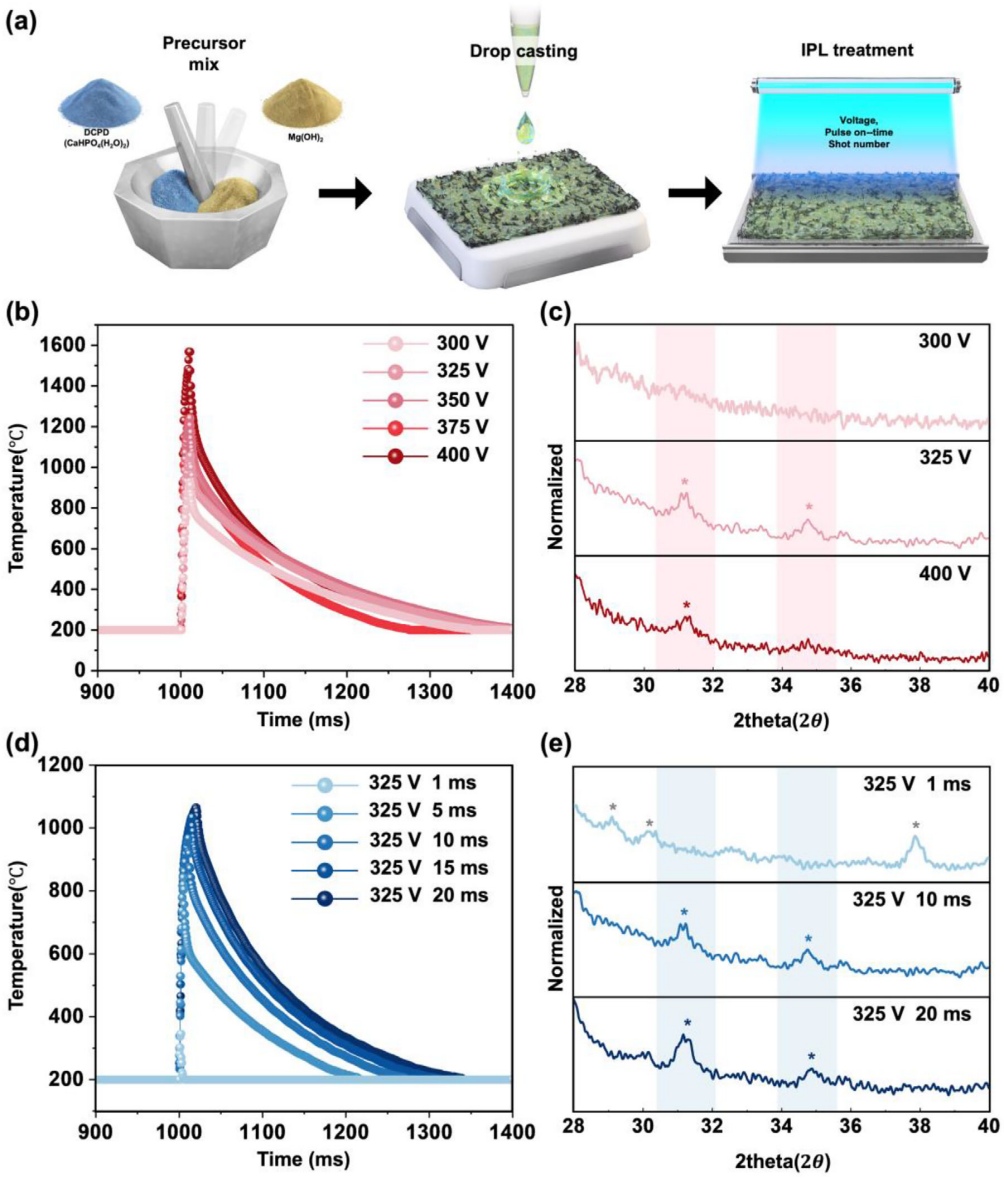
Optimization of IPL conditions for whitlockite synthesis (voltage and pulse duration). (a) Synthesis process of IPL whitlockite. (b) Temperature‐time curves with applied voltage conditions 300, 325, 350, 375, and 400 V. (c) GIXRD pattern with voltage conditions 300, 325, and 400 V. (d) Temperature–time curve with increasing pulse on‐time 1 ms to 20 ms at 5 ms steps. (e) GIXRD pattern with pulse on‐time regulation of 1, 10, and 20 ms.

We first examined whitlockite formation under different IPL power conditions. The IPL can be controlled by adjusting the voltage (300–500 V), pulse on–time (1–20 ms), and number of shots, which regulate the synthesis temperature, heating/cooling rate, and heating pulses, respectively. Figure 1b presents a temperature–time curve with voltage control, clearly illustrating the ultrafast heating and cooling properties. An infrared thermography camera detects surface temperatures as high as 1600°C during IPL irradiation. The temperature peak increased with increasing voltage from 300 V to 400 V in 25 V increments. At 300 V, the temperature was 947°C, at 325 V, around 1056°C, at 350 and 375 V, over 1200°C, and at 400 V, close to 1600°C. The synthetic temperature (IPL voltage) dependent inorganic phase change was then investigated by Grazing Incidence x‐ray Diffraction (GIXRD) for each sample (Figure S1). In Figure 1c, no crystalline peaks were observed at 300 V. In contrast, samples processed above 325 V (>1000°C) showed characteristic whitlockite peaks around 31.2 ^○^ and 34.8 ^○^. These correspond to the two most intense whitlockite peaks and are in good agreement with previous reports, different from other calcium phosphates [1]. Unfortunately, other weaker peaks were not clearly detected, likely due to strong substrate signals, the fine size of our whitlockite, and background noise. Actually, the Ca‐ Mg‐phosphate system has low eutectic point at 1150–1200°C as opposed to other ceramics [32]. So, it can be supposed that the precursor could be a localized liquid phase and gained ion diffusion between two precursors, finally forming a new Ca‐Mg‐phosphate phase. Interestingly, 400 V shows that the peaks were offset when the temperature exceeded a certain level. This behavior may originate from the intense thermal shock imparted by IPL, where excessive local heating can cause precursor decomposition [33] or micro‐explosions [34] at the substrate, thereby reducing peak intensity. As a result, it indicates that the IPL temperature condition has a trade‐off in the formation of whitlockite.

Subsequently, whitlockite phase formation was also confirmed by varying the pulse on–time while fixing the voltage conditions at 325 V, under which the whitlockite phase was formed. As shown in Figure 1d, the temperature peak gradually increased as the pulse on–time increased by 5 ms from 1 to 20 ms (max). Despite the same voltage, 1 ms reached only around 345°C, and 5 ms also reached around 842°C. For pulse on‐time of 10 ms and above, it was confirmed that the temperature increased consistently to around 1100°C. Consequently, the phase change in the GIXRD pattern with pulse on‐time was investigated (Figure S2).

As can be seen in Figure 1e, the precursor CaHPO_4_·2H_2_O and Mg(OH)_2_ peaks were present at 1 ms, with no detectable whitlockite peak. At 5 ms, both precursor and whitlockite peaks appeared, implying that the phase transformation occurred (Figure S2b). From 10 ms, peaks around 31.2 ° and 34.8 ° were observed, and these peaks sharpened further at 20 ms, suggesting enhanced crystallinity with longer pulse duration (Figure 1e). These results highlight that enough heating and cooling time is a critical factor in the formation and growth of whitlockite phases, as precursors undergo partially melting and phase transformation during IPL treatment. These observations indicate that whitlockite formation occurs within specific IPL conditions, where the appropriate temperature range and pulse duration are essential for stabilizing the phase.

### 2.3 Effect of IPL Shot Number on Whitlockite Phase Evolution and Crystallization Process

This IPL treatment process provides very fast heating and cooling rates compared to conventional wet chemistry synthesis methods. As shown in Figure 2a, the IPL process achieves heating (∼43 000 K S^−1^) and cooling rates (∼2700 K S^−1^) that are several thousand times faster than those of traditional heat treatment methods (< 1 K S^−1^). This ultrafast thermal cycling prevents excessive grain growth [35], phase separation, and particle size inhomogeneity during inorganic synthesis [36, 37], thereby enabling the rational formation of metastable whitlockite. We further investigated the phase of whitlockite by controlling the IPL shot number, defined as the number of IPL irradiation repetitions. Figure 2b demonstrates that the temperature peak stabilized near 1100°C, independent of the shot number adjusted from 1 to 20 shots. Distinct whitlockite peaks were also detected at 2θ ≈ 31.2° (2 1 7) and 34.8° (2 2 0) by GIXRD (Figure 2c). In a magnified view of the 28 ° to 40 ° region, we could confirm that a whitlockite peak became more pronounced after 10 shots compared to a single shot (Figure 2d). We found that a new phase emerged only when a mixed precursor of DCPD and Mg(OH)_2_ was subjected to IPL treatment, whereas each precursor treated individually showed only its dehydrated form (Figure S3) [38, 39]. These findings suggested that repeated IPL irradiations promote phase transformation of precursors on the carbon substrate. Comparative GIXRD analysis between conventionally synthesized and IPL‐synthesized whitlockite confirmed that the newly formed phase corresponds to whitlockite phase (Figure S4a), which was further corroborated by Fourier transform infrared spectroscopy (FTIR) analysis confirming the characteristic HPO_4_^2^^−^ stretching mode. (Figure S4b) [40].

**FIGURE 2:**
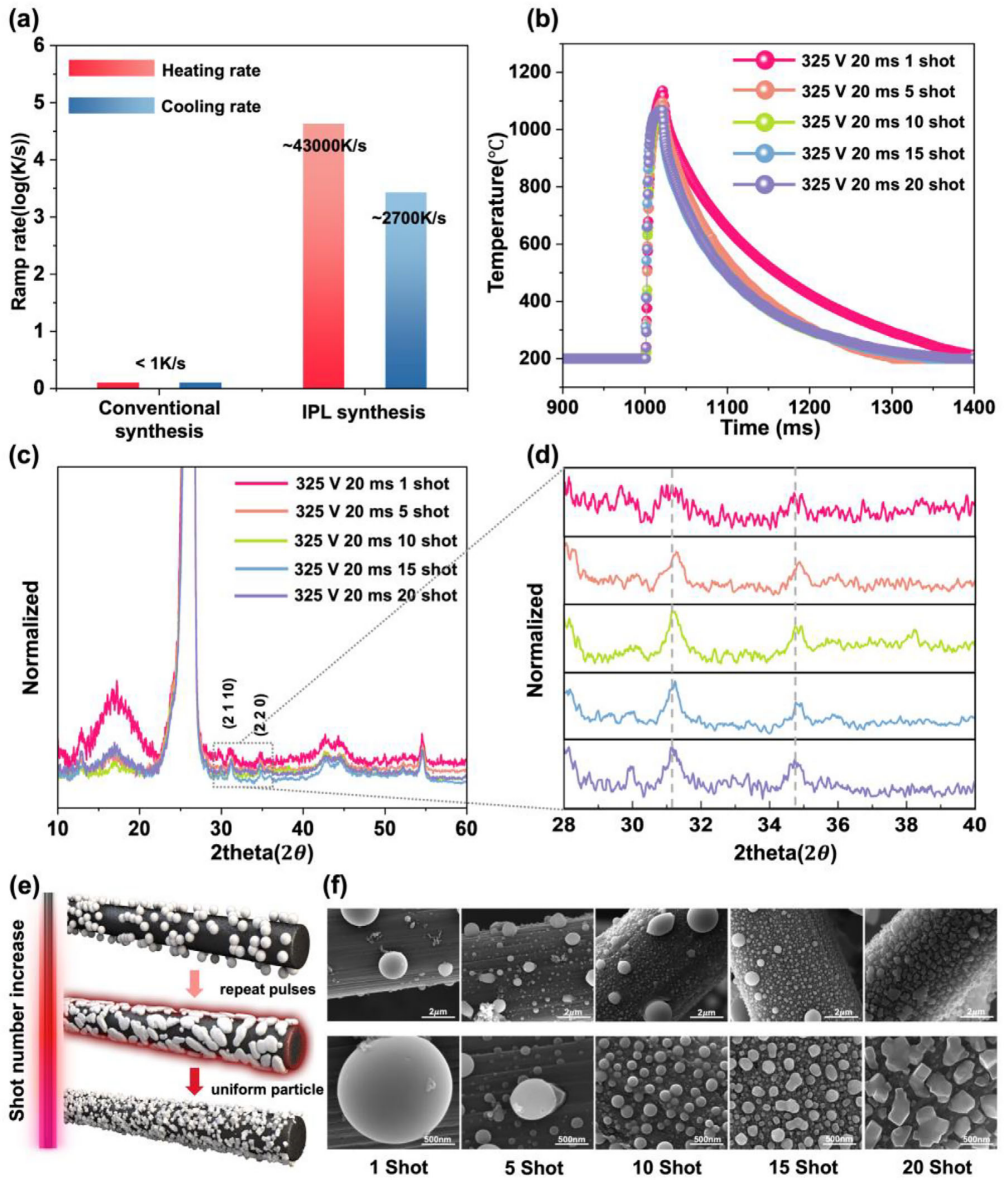
Effect of IPL shot number on whitlockite phase evolution and crystallization process. (a) Comparison of heating and cooling rate (K/s) between IPL synthesis and the conventional method. (b) Temperature (^○^C)‐Time (msec) curves with different shot numbers of IPL. (c) GIXRD patterns showing the evolution of whitlockite peaks with increasing shot number. (d) Enlarged GIXRD patterns highlighting the whitlockite peaks of each sample. (e) Illustration of particle attachment and crystallite growth on carbon fiber with increasing shot number. (f) FE–SEM images confirmed the development of uniform crystals on carbon fibers.

To further investigate the effect of the number of IPL shots, the ex‐situ TEM analysis was carried out. Figure S5a illustrates that the precursor on carbon fibers underwent repeated transient liquid phase formation and subsequent ion diffusion with pulsed thermal cycling, leading to new phase formation. In Figure S5a, (I) shows that with a small number of shots, precursors underwent insufficient melting and partially transformed into a liquid phase. In contrast, (II) demonstrated that with enough shots, complete exchanging Mg^2+^ and Ca^2+^ ions and new phase formation occurred, leading to the formation of a uniform whitlockite phase. As shown in Figure S5b, a single‐shot IPL‐treated particle displayed a heterogeneous distribution of Ca, Mg, P, and O, with a size of around 2 µm. The SAED pattern revealed a heterogeneous multiphase intermediate, indicating mixed phases. After 10 shots, elemental distribution became more homogeneous, and the particle size was 1.47 µm. Similar to the first shot, the SAED pattern confirmed a partially transformed phase, but the pattern itself was clearer, suggesting improved crystallinity. At 20 shots, the particle exhibited a uniform rhombohedral whitlockite structure with a reduced size of ∼250 nm. Average size was confirmed sub‐200 nm through SEM images. (Figure S5c) SAED pattern further confirmed the whitlockite peaks (2 1 4) and (2 2 0), and the latter of which was also identified through GIXRD. This suggests that a transient liquid phase precursor was initially formed through incomplete dehydration. As the number of shots increased, particle fragmentation occurred to release trapped H_2_O vapor, followed by gradual ion diffusion and stabilization of the new phase (Figure S5d).

Figure 2e schematically depicts the interfacial evolution between the precursor and microheater carbon fiber during whitlockite formation. The precursor attached to the fiber spherical aggregates at first, which likely reflects poor wettability between the precursor and carbon substrate. Under such conditions, a transient liquid phase would tend to adopt a spherical morphology to minimize surface energy. This interpretation is consistent with the FE–SEM observations in Figure 2f. After a single shot, melted precursors formed into large spherical shapes, whereas repeated irradiation gradually reduced the particle size and produced a more uniform morphology. Such evolution suggests that the precursors did not transform in a single step, but instead underwent repeated transient liquid‐like restructuring during successive IPL shots. A possible explanation is that fragmentation by the release of trapped H_2_O vapor can increase surface area and facilitate subsequent ion diffusion and crystallization. In addition, defect‐rich regions on the carbon fiber may provide thermodynamically favorable sites for heterogeneous nucleation [20, 41]. In contrast, carbon fiber‐unexposed regions similarly exhibited spherical aggregate morphology, yet showed no crystalline facet development (Figure S6), confirming that direct carbon fiber contact is essential for complete whitlockite crystallization. Raman analysis (Figure S7) suggests that repeated IPL treatment increases the defect density in the carbon substrate, which may further promote nucleation on the carbon fiber surface. During 1 to 10 shots, the precursor mixture repeated transient liquid phase formation with local H_2_O vapor generation and fragmentation, accompanied by gradual ion diffusion and Mg^2+^ substitution at Ca^2+^ ion sites. These substitutions are expected to induce local lattice distortion, thereby stabilizing the whitlockite phase. After 20 shots, the particle morphology exhibited facets of rhombohedral whitlockite structure, suggesting that repeated thermal cycling promoted precursor dehydration followed by transient liquid phase mediated ion diffusion, and subsequent crystallization. Collectively, these results confirmed the formation of the whitlockite phase by IPL treatment, with phase emergence governed by the IPL conditions such as voltage, pulse on–time, and shot number. An operating window near 1100°C with sufficient irradiation duration is required for nucleation and growth.

### 2.4 Role of Localized H_2_O Vapor Pressure in Whitlockite Formation From Hydrated Precursors

In the conventional thermal annealing process, HPO_4_^2−^ containing in whitlockite undergoes a gradual dehydration and structural rearrangement, transforming into other calcium phosphate phases such as TCMP (magnesium–substituted β–TCP) [42, 43]. In contrast, our IPL system generates a transient high temperature of approximately 1100°C and thus provides a reaction environment distinct from conventional annealing. Indirect experimental assessment of H_2_O vapor pressure effects under bulk confinement conditions suggests that localized vapor pressure plays a critical role in governing precursor transformation kinetics (Figure S8). We hypothesize that rapid precursor dehydration and the local H_2_O vapor pressure at the precursor‐substrate interface was contribute to the retention of HPO_4_^2−^ within the whitlockite structure by delaying its complete dehydration and decomposition under ultrafast high temperature conditions.

In Figure 3a, we suggested a phase formation tendency of partial pressure of H_2_O (X‐axis) and temperature (Y‐axis). This tendency was confirmed through the FactSage program and calculated by an arbitrary thermodynamic database of whitlockite (Figure S9). At a low partial pressure of H_2_O/bar, no stability field for whitlockite is predicted over the entire temperature range ((I) region). As the partial pressure of H_2_O increased, the whitlockite began to appear together with other calcium‐ and magnesium‐phosphate phases ((II) region). In particular, when the temperature and partial pressure of H_2_O reach a high enough point, the whitlockite phase is dominant ((III) region). These trends indicate that whitlockite formation is favored under high H_2_O partial pressure and within a specific temperature window. This trend is consistent with previous reports describing the relationship between protonated phosphate species and water‐rich environments [44, 45]. In hydroxyapatite‐based systems, thermal dihydroxylation has been shown to depend on the water vapor partial pressure, and the introduction of water vapor can suppress dihydroxylation and thermal decomposition [31]. This concept supports the idea that an H_2_O‐rich local environment may reduce the driving force for dehydration of protonated phosphate units.

**FIGURE 3:**
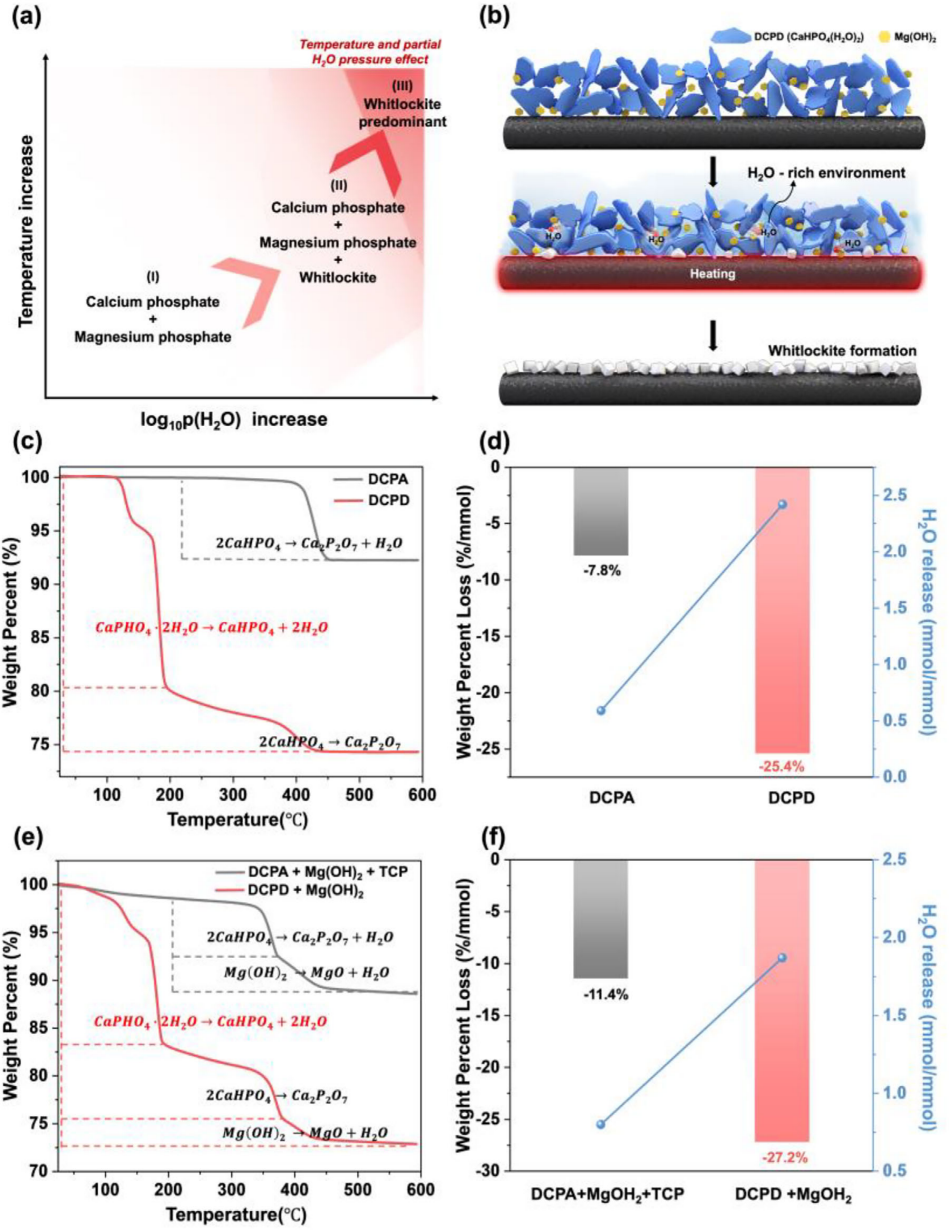
Role of localized H_2_O vapor pressure in whitlockite formation from hydrated precursors. (a) The tendency of phase formation is illustrated according to H_2_O partial pressure (X–axis) and temperature (Y–axis). (b) Scheme during IPL processing, rapid heating dehydrates the hydrate precursor, and transient H_2_O is confined at the precursor‐carbon interface (c, e). Thermogravimetric analysis (TGA) of calcium phosphate DCPA (Monetite, CaHPO_4_; Anhydrous) and DCPD (Brushite, CaHPO_4_·2H_2_O; Hydrate) and Anhydrous precursor (DCPA + TCP + Mg(OH)_2_) and hydrate precursor (DCPD + Mg(OH)_2_). (d, f) Calculation of H_2_O release mmol from the TGA data for each composition.

Based on this hypothesis, we propose that the interaction between microheating fibers and hydrated precursors generates a momentary H_2_O‐rich and high‐pressure microenvironment, favorable for whitlockite formation. As illustrated in Figure 3b, the process begins with precursors deposited on a carbon substrate, followed by IPL exposure, and concludes with the crystallized whitlockite phase. In the middle of Figure 3b, the precursors on the substrate transiently melt, generating H_2_O with the dehydration of the precursor during IPL treatment. Densely packed powders on the carbon fiber microheater restrict the escape of evolved H_2_O, which becomes trapped at the precursor‐carbon substrate interface. This transiently interface‐confined, high‐pressure, elevated temperature, H_2_O‐rich microenvironment promotes and stabilizes whitlockite formation. Consequently, at the end of Figure 3b, we get highly ordered whitlockite particles. Therefore, we hypothesize that results in the formation of a localized H_2_O partial pressure environment through the ultrafast thermal shock can bias the reaction pathway toward whitlockite.

To clarify this assumption, we evaluated the difference in H_2_O release by precursor type. We performed TGA to compare DCPA (CaHPO_4_) and DCPD (CaHPO_4_·2H_2_O) among calcium phosphates. As shown in Figure 3c, both materials exhibit progressive mass loss up to 600°C. DCPA undergoes a principal decomposition into pyrophosphate at near ∼400°C, whereas DCPD (hydrate form) displays two distinct events: a primary dehydration at 173.5–177.2°C yielding CaHPO_4_ + 2H_2_O, followed by a second step near ∼400°C associated with conversion of DCPA to pyrophosphate [46]. Figure 3d quantifies the mass loss and the amount of H_2_O for an equal initial molar amount. DCPA shows a 7.8% weight percent loss, while DCPD shows a 25.4% weight percent loss. The corresponding H_2_O produced was 0.59 mmol for DCPA and 2.42 mmol for DCPD—approximately 4.1‐fold difference. This result was consistent with thermodynamic calculations of DCPD and DCPA (Figure S10), indicating that our synthesis conditions are favorable for the dehydration of precursors.

And then, TGA analysis was conducted using a precursor mixture matching that used in the IPL synthesis. The precursor composition of the DCPA + TCP + Mg(OH)_2_ precursor was selected based on the prior solid‐state synthesis method of whitlockite [12]. For convenience, this precursor is denoted as the anhydrous precursor, whereas the DCPD + Mg(OH)_2_ precursor is denoted as the hydrated precursor. Figure 3e compares the mass‐loss profiles of the two precursor mixtures as a function of temperature. In addition to the decomposition process described in Figure 3e, the precursor mixtures also exhibit a decomposition process of Mg(OH)_2_. Anhydrous precursor shows an 11.4 wt.% loss, whereas the hydrated precursor shows a 27.2 wt.% loss (Figure 3f). On a per‐mole basis, the amount of H_2_O evolved from the hydrated mixture was 2.3 times that of the anhydrous mixture. These results show that H_2_O yield depends sensitively on the precursor types. Ultimately, the precursor dehydration induces a transient H_2_O‐rich and high‐pressure environment that stabilizes the whitlockite phase.

### 2.5 Precursor‐Dependent Phase Evolution During IPL Processing

Subsequently, investigated whether a transient H_2_O‐rich, high‐pressure environment influenced whitlockite formation. Figure 4a,b show GIXRD results of the anhydrous and hydrated precursors after IPL treatment under identical conditions. In both cases, the original calcium phosphate (DCPA, TCP, DCPD) peak (blue circle) and magnesium hydroxide (Mg(OH)_2_) peak (yellow rhomb) disappeared after IPL, while a new whitlockite peak (red pentagon) emerged. These results indicate that rapid thermal cycling during IPL induced the transient melting of both precursors, leading to phase transformation. Although both samples exhibited XRD peaks corresponding to the whitlockite phase, these reflections were nearly indistinguishable from those of β − TCP or TCMP [47]. Therefore, the presence of the HPO_4_^2−^ group was considered a crucial fingerprint for identifying whitlockite [48, 49]. Consequently, XPS analysis (Figure 4c,d) revealed clear differences between precursor types. For the anhydrous precursor, the P2p peak shifted to the right after IPL treatment (Figure S11a) and peak fitting identified only a single PO_4_^3−^ bonding (Figure 4c). In contrast, for the hydrate precursor, the P2p peak shift was minimal or slightly to the left (Figure S11b). Peak fitting resolved both the PO_4_^3−^ (65.5%) at 133.0 eV and HPO_4_^2−^ (34.4%) at 134.0 eV (Figure 4d) [50, 51]. Since whitlockite is characterized by the HPO_4_^2−^ group, it was ultimately demonstrated that the whitlockite phase was well defined from the hydrate precursor. Thus, the instantaneous dehydration of the hydrate precursor and resulting localized H_2_O‐rich and high‐pressure environment were likely key factors in promoting whitlockite formation by retaining HPO_4_^2−^ structure under transient high‐temperature conditions. Therefore, we suggest that whitlockite formation via IPL can be attributed to the unique environment generated by this transient and confined interfacial pocket. Compared with previously reported whitlockite synthesis, this method is significantly faster and enables the production of nanoparticles (Figure 4e). Conventional wet chemical methods can produce nano‐sized and uniform particles, but they rely on complex, time‐consuming multistep procedures [1, 5, 8, 43, 49, 50, 52, 53, 54, 55]. Microwave‐assisted [56, 57] and solid‐state synthesis [12, 58] shorten the synthesis speed through mechanical vibration and thermal, respectively, yet they often give broad size distributions and struggle to reach the nanoscale. A more detailed comparison of synthesis methods is provided in Table S1. By contrast, IPL couples ultrafast photothermal heating with interfacial microheating, enabling rapid synthesis while forming nanoscale crystallites. Beyond its synthetic advantages, the IPL‐synthesized whitlockite demonstrated excellent biocompatibility and cell viability (Figure S12a), confirming its biological stability and opening up the possibility as a promising platform for the development of next‐generation bone regeneration bioceramics. (Figure S12b)

**FIGURE 4:**
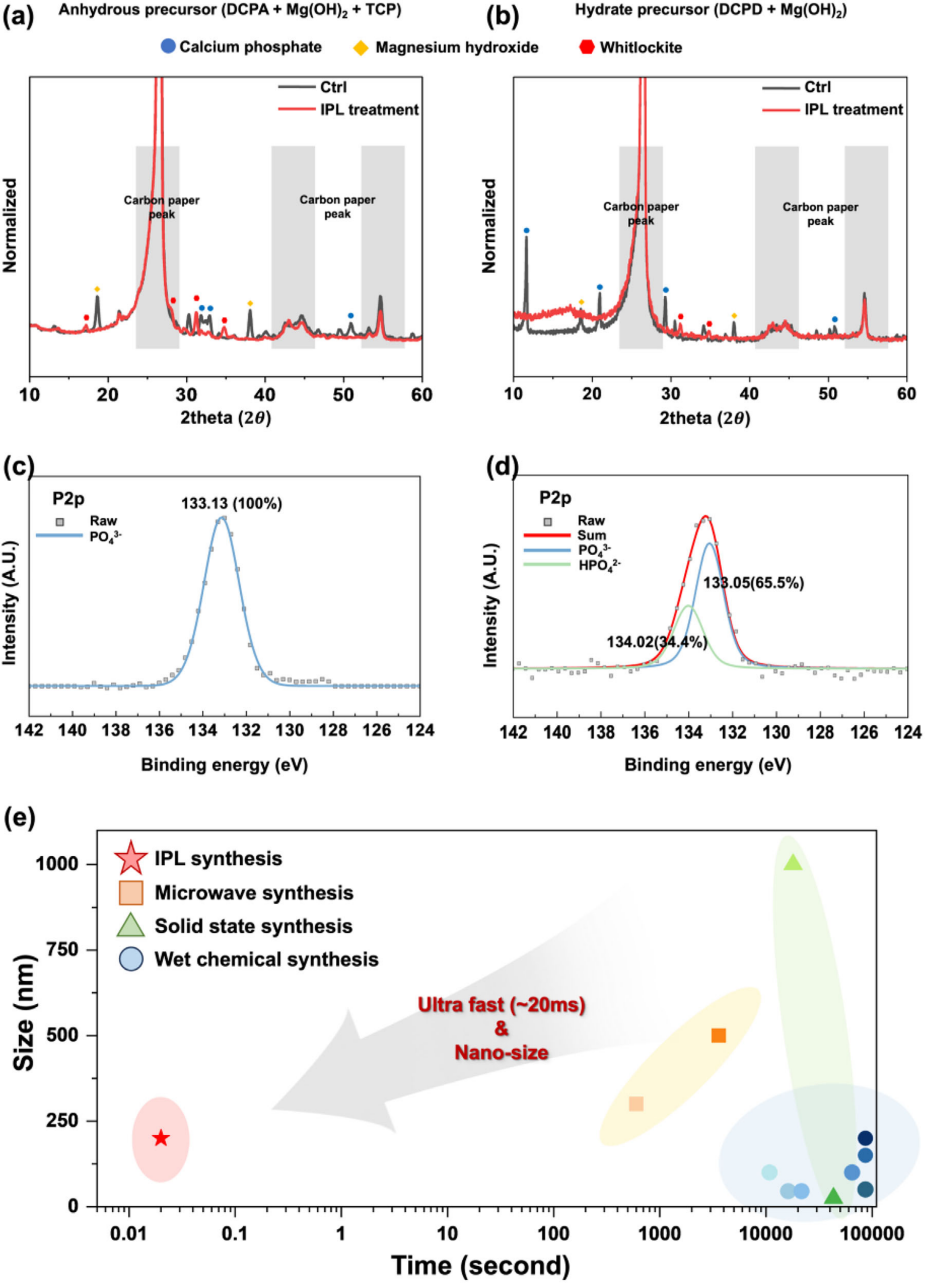
Precursor‐dependent phase evolution during IPL processing. (a, b) XRD patterns showing peak changes before and after IPL treatment. (c, d) XPS analysis revealing that the anhydrous precursor lacked HPO_4_^2−^ bonding, while the hydration precursor exhibited HPO_4_^2−^ bonding of whitlockite. (e) Comparison of synthesis time between the conventional methods and IPL synthesis.

## 3 Conclusions

We demonstrate an efficient approach for whitlockite synthesis through a simple, rapid, and powerful IPL heat treatment. Phase formation was clearly verified by GIXRD under varying voltages, pulse on‐time, and shot number. This process achieved heating (∼43 000 K S^−1^) and cooling (∼2700 K S^−1^) rates—orders of magnitude faster than conventional thermal methods(∼1K S^−1^). Increasing the number of IPL shots enhanced the whitlockite XRD intensity and produced denser, more uniform particles, as confirmed by SEM and TEM–EDS mapping. Furthermore, SAED analysis under 325 V, 20 ms, and 20 shots confirmed the whitlockite crystal structure, with elemental compositions consistent with conventionally synthesized whitlockite.

Beyond IPL processing parameters, precursor composition played a critical role in whitlockite phase formation. The ultrafast thermal cycle of the IPL induced instantaneous dehydration of the hydrate precursor, generating a transient H_2_O‐rich, high‐pressure environment that may have promoted whitlockite formation by facilitating the retention of HPO_4_^2−^. Phase diagram analysis further supported the possibility that elevated H_2_O partial pressure at high temperatures could favor whitlockite phase stability. This proposed mechanism is supported by TGA quantified H_2_O release, XPS identified HPO_4_^2^^−^ bonding, and FTIR analysis confirming characteristic HPO_4_^2^^−^ absorption bands.

In summary, these findings establish IPL as a powerful platform for the instantaneous synthesis of whitlockite, effectively overcoming the limitations of conventional routes. This approach not only enables nanoscale particle formation within milliseconds but also opens new pathways for developing advanced calcium phosphate‐based bioceramics. With further biological validation, including comprehensive in vitro osteogenic evaluation and in vivo bone regeneration studies, IPL‐driven whitlockite synthesis holds strong potential to emerge as a next‐generation platform for bone regeneration applications.

## 4 Experimental Section/Methods

### 4.1 IPL Whitlockite Sample Preparation

#### 4.1.1 Materials

DCPD (CaHPO_4_·2H_2_O) and DCPA (CaHPO_4_) were prepared from calcium hydroxide (Ca(OH)_2_, ≥ 96%, Sigma–Aldrich) and phosphoric acid (H_3_PO_4_ 85 wt% in H_2_O, Sigma–Aldrich) following a reported procedure. Magnesium hydroxide (Mg(OH)_2_, reagent grade, 95%, Sigma–Aldrich) and calcium phosphate tribasic (TCP, Ca_3_(PO_4_)_2_,  practical grade REAGENTS DUCSAN) were used as received. Carbon paper substrates were Avcarb GDS P50 carbon fiber papers (20 × 20 cm square; AvCarb Material Solution, Nara Cell–Tech). Glass slides (LK LAPKOREA) and pre‐cleaned microscope slides (MATSUNAMI) were used to cover samples during IPL.

#### 4.1.2 Precursor Powder Preparation

The anhydrous precursor was a mixture of DCPA, TCP, and Mg(OH)_2_ = 1 :  0.625 :  1.35 (wt.% ratio) according to a solid‐state whitlockite recipe [12]. The hydrate precursor was prepared by mixing DCPD and Mg(OH)_2_ = 4 : 1 (wt.% ratio). Each mixture (1.0 g) was wet mixed with ethanol (1 mL) in a mortar and pestle until all the solvent evaporated completely, then stored at room temperature.

#### 4.1.3 Sample Preparation

A suspension was prepared by dispersing the mixed precursor (10 mg mL^−1^) in ethanol (100 mg powder in a 20 mL vial). The slurry was tip‐sonicated for 3 min (pulse 20 s on/30 s off). Carbon paper (1×2 cm) placed on a 63.5°C hot plate was drop‐cast three times with 100 µL aliquots. After complete solvent evaporation, the substrate was fixed between two glasses for IPL processing.

### 4.2 Intense Pulsed Light Irradiation Conditions

An intense pulsed light (IPL) system (Photocura, PLT) equipped with a xenon lamp (First Light Tech.) emitting a continuous broad spectrum covering the UV–Vis–NIR region was used as the irradiation source. Process parameters were voltage, pulse on‐time, and shot number (pulse counts); the lamp‐sample distance (1.7 cm) was kept constant. Voltage was varied from 300 to 400 V in 25 V increments. Pulse on–time was increased in 5‐ms steps (1–20 ms). For each condition, the number of shots was adjusted as specified in the text. Thermal uniformity of the carbon substrate was confirmed by repeatable experiments. (Figure S13)

### 4.3 Characterizations

X‐ray diffraction (XRD) was performed on a Rigaku D/MAX–220 using Cu Kα radiation (λ = 1.5406 Å). Grazing‐incidence XRD (GIXRD) was collected at a fixed incident angle ω = 1°, with a scan rate of 2°min^−^^1^ and a step size of 0.02°. Field‐emission scanning electron microscopy (FE‐SEM, Inspect F50, FEI; Schottky FEG) images were acquired at 10 kV, spot size 3.0, and a working distance of 10 mm. Also, samples were Pt‐coated (E–1045, HITACHI; 15 mA, 30 s) before imaging. Transmission electron microscopy with EDS mapping (Talos F200X, FEI; Super‐EDX) and selected‐area electron diffraction (SAED) was used to determine microstructure and composition. D‐spacings from SAED were measured with the Image J program. I_d_/I_g_ ratio was evaluated by using a Raman microscope (inVia, Renishaw, UK) with a 532 nm laser wavelength and a 5 mW incident laser power. Average size analysis was conducted using ImageJ software on SEM images (*n* = 60; 3 independent samples, 20 particles per sample). The surface chemical bonding states were analyzed by x‐ray photoelectron spectroscopy (XPS, PHI 5000 VersaProbe, ULVAC–PHI) with and an Al Kα source (hν = 1486.6 eV). All spectra were charge‐corrected to the adventitious C 1s peak at 284.6 eV. Peak fitting was performed using CasaXPS software with a Shirley‐type background subtraction. The P 2p spectra were deconvoluted into two components, PO_4_^3^^−^ (133.1 eV) and HPO_4_^2^^−^ (134.0 eV), with peak assignments referenced to previously reported XPS characterization of whitlockite phases [50, 51]. Fourier transform infrared spectroscopy (Nicolet iS20 FTIR Spectrometer, Thermo Fisher Scientific) measurements were performed with a diamond ATR crystal. All spectra were collected over the range of 500–4000 cm^−1^ at a resolution of 8 cm^−1^ with 32 scans per sample. Bare carbon paper was used as the background reference for all measurements, and three independent regions were measured per sample to confirm spatial consistency. Thermogravimetric analysis (TGA, TGA 550, TA Instruments) was carried out on dry powders from 25 to 600°C at 10.0°C min^−^^1^ to quantify mass loss and H_2_O evolution.

### 4.4 FactSage Calculation

Thermodynamic calculations were performed using FactSage 8.3 software. DCPD–Mg(OH)_2_–H_2_O phase diagram (Figure S9) was constructed using the well‐available thermodynamic private database for the Ca─Mg‐P─O─H system, except for a whitlockite phase, for which Gibbs energy was unknown and roughly determined in this study. The calculation was performed at a DCPD to Mg molar ratio of 9:1, while the partial pressure of H_2_O (log_P_(H_2_O)/bar) was varied from −5 to 3. For Figure S10, the temperature‐dependent transformations of DCPD and DCPA were calculated using the Equilib program, assuming 1 mol of each substance at 1 atm.

### 4.5 Cell Viability Assay

Eluates were prepared in accordance with ISO 10993‐12 at an extraction ratio of 6 cm^2^/mL. IPL_whitlockite, bare carbon paper, and IPL‐treated carbon paper (w/o precursor) were immersed in serum‐free α‐MEM at 37°C for 1, 2, and 3 days, respectively. For the CCK‐8 assay, MC3T3‐E1 cells were seeded in 4‐well plates (1 cm × 2 cm, *n* = 4) at a density of 1 × 10^4^ cells/well and cultured for 24 h. The medium was then replaced with 100% eluate for an additional 24 h, after which cell viability was quantified using CCK‐8 (Dozen Bio, Korea) at 450 nm and expressed as a percentage relative to the untreated control. For Live/Dead staining, MC3T3‐E1 cells were seeded directly onto the substrate and cultured for 24 h, followed by staining with a Live/Dead Viability/Cytotoxicity Kit (Dozen Bio, Korea) and fluorescence imaging. For morphological observation, cell‐seeded substrates were dehydrated in 90% ethanol, dried, and examined by portable SEM

## Funding

This work was supported by the Technology Development Program (RS‐2024‐00506838) and the Collabo R&D Program (RS‐2025‐02315709) funded by the Ministry of SMEs and Startups (MSS, Korea)

## Conflicts of Interest

The authors declare no conflicts of interest.

## Supporting information




**Supporting File**: advs76175‐sup‐0001‐SuppMat.docx.

## Data Availability

The data that supports the findings of this study are available in the supplementary material of this article.
