# Supplementary material for: Non‐Equilibrium Synthesis of Whitlockite Assisted by Localized H2O Vapor Pressure
Source: Adv Sci (Weinh). 2026 Jun 16;13(55):e76175. doi: 10.1002/advs.76175 (PMC13336570; doi:10.1002/advs.76175)
Supplement: Supplementary file 1 [file ADVS-13-e76175-s001.docx]

Supporting Information

**Non-equilibrium Synthesis of Whitlockite Assisted by Localized H_2_O vapor pressure**

Min-Jung Kim^1,2^, Min-woo Lee^3^, In-Ho Jung^2^, Seung-Kyun Kang^2,*^, Ji-Soo Jang^4,*^, Hyung-Seop Han^1,*^

**Supporting figures**

1. XRD analysis following voltage conditions ∙∙∙∙∙∙∙∙∙∙∙∙∙∙∙∙∙∙∙∙∙∙∙∙∙∙∙∙∙∙∙∙∙∙∙∙∙∙∙∙∙∙∙∙∙∙∙∙∙∙∙∙∙∙∙∙∙∙∙∙∙∙∙∙∙ P.3
2. XRD analysis following pulsed on time conditions ∙∙∙∙∙∙∙∙∙∙∙∙∙∙∙∙∙∙∙∙∙∙∙∙∙∙∙∙∙∙∙∙∙∙∙∙∙∙∙∙∙∙∙∙∙∙∙∙∙∙∙∙∙ P.4
3. XRD patterns under different precursor conditions ∙∙∙∙∙∙∙∙∙∙∙∙∙∙∙∙∙∙∙∙∙∙∙∙∙∙∙∙∙∙∙∙∙∙∙∙∙∙∙∙∙∙∙∙∙∙∙∙∙∙∙∙∙∙ P.5
4. Structural characterization of IPL synthesized whitlockite ∙∙∙∙∙∙∙∙∙∙∙∙∙∙∙∙∙∙∙∙∙∙∙∙∙∙∙∙∙∙∙∙∙∙∙∙∙∙∙∙∙∙ P.6
5. TEM–EDS mapping images and SAED pattern analysis ∙∙∙∙∙∙∙∙∙∙∙∙∙∙∙∙∙∙∙∙∙∙∙∙∙∙∙∙∙∙∙∙∙∙∙∙∙∙∙∙∙∙∙∙∙ P.7
6. Bulk SEM images of carbon paper ∙∙∙∙∙∙∙∙∙∙∙∙∙∙∙∙∙∙∙∙∙∙∙∙∙∙∙∙∙∙∙∙∙∙∙∙∙∙∙∙∙∙∙∙∙∙∙∙∙∙∙∙∙∙∙∙∙∙∙∙∙∙∙∙∙∙∙∙∙∙∙∙∙∙∙∙∙∙∙ P.8
7. Raman spectral analysis under different samples under in same IPL irradiation conditions∙∙∙∙∙∙∙∙∙∙∙∙∙∙∙∙∙∙∙∙∙∙∙∙∙∙∙∙∙∙∙∙∙∙∙∙∙∙∙∙∙∙∙∙∙∙∙∙∙∙∙∙∙∙∙∙∙∙∙∙∙∙∙∙∙∙∙∙∙∙∙∙∙∙∙∙∙∙∙∙∙∙∙∙∙∙∙∙∙∙∙∙∙∙∙∙∙∙∙∙∙∙∙∙∙∙∙∙∙∙∙∙∙∙∙∙∙∙∙ P.9
8. Effect of H2O vapor confinement on IPL induced phase transformation ∙∙∙∙∙∙∙∙∙∙∙∙∙∙∙∙∙∙ P.10
9. Phase diagram calculation of CaHPO_4_–Mg(OH)_2_–H_2_O ∙∙∙∙∙∙∙∙∙∙∙∙∙∙∙∙∙∙∙∙∙∙∙∙∙∙∙∙∙∙∙∙∙∙∙∙∙∙∙∙∙∙∙∙∙ P.11
10. Equilibrium calculation of DCPA(CaHPO_4_) and DCPD(CaHPO_4_(H_2_O)_2_) ∙∙∙∙∙∙∙∙∙∙∙∙∙∙∙∙ P.12
11. XPS analysis before IPL treatment was performed for each precursor ∙∙∙∙∙∙∙∙∙∙∙∙∙∙∙∙∙∙∙∙∙∙ P.13
12. In vitro cytocompatibility evaluation of IPL synthesized whitlockite ∙∙∙∙∙∙∙∙∙∙∙∙∙∙∙∙∙∙∙∙∙∙∙∙ P.14
13. Thermal uniformity of IPL system ∙∙∙∙∙∙∙∙∙∙∙∙∙∙∙∙∙∙∙∙∙∙∙∙∙∙∙∙∙∙∙∙∙∙∙∙∙∙∙∙∙∙∙∙∙∙∙∙∙∙∙∙∙∙∙∙∙∙∙∙∙∙∙∙∙∙∙∙∙∙∙∙∙∙∙∙∙ P.15
14. Table 1∙∙∙∙∙∙∙∙∙∙∙∙∙∙∙∙∙∙∙∙∙∙∙∙∙∙∙∙∙∙∙∙∙∙∙∙∙∙∙∙∙∙∙∙∙∙∙∙∙∙∙∙∙∙∙∙∙∙∙∙∙∙∙∙∙∙∙∙∙∙∙∙∙∙∙∙∙∙∙∙∙∙∙∙∙∙∙∙∙∙∙∙∙∙∙∙∙∙∙∙∙∙∙∙∙∙∙∙∙∙∙∙∙∙∙∙∙∙∙∙∙ P.16

FIGURE S1. XRD analysis following voltage conditions

(a-e) GIXRD patterns of samples irradiated at 300 V, 325 V, 350 V, 375 V, and 400 V, respectively, showing enhanced whitlockite phase crystallinity at intermediate voltage.

FIGURE S2. XRD analysis following pulsed on–time conditions

(a-e) GIXRD patterns of samples irradiated at 325 V 1 ms, 5 ms, 10 ms, 15 ms, and 20 ms, respectively. Enhanced whitlockite phase crystallinity was observed with increasing pulsed on-time.

FIGURE S3. XRD patterns under different precursor conditions

Bare Carbon paper denotes the untreated carbon substrate, while Carbon paper_IPL denotes the carbon substrate after IPL treatment only. DCPD_IPL and Mg(OH)_2__IPL denote the individual CaHPO_4_·2H_2_O and Mg(OH)_2_ precusors after IPL irradiation, respectively. DCPD+Mg(OH)2_IPL represents the original mixed-precursor condition used for whitlockite synthesis.

FIGURE S4. Structural characterization of IPL-synthesized whitlockite

(a) Comparative GIXRD patterns of conventionally synthesized whitlockite (Conventional_WH) and IPL-synthesized whitlockite (IPL_WH) on carbon paper substrate. (b) FTIR spectra of the precursor mixture, Conventional_WH, and IPL_WH cast on identical carbon paper substrates, with bare carbon paper used as the background reference. HPO₄²⁻ form blue-shifting hydrogen bonds with these carbon surface functional groups, leading to increased electron density at the P-O-H bond and a consequent blue shift in the absorption frequency.[1]

FIGURE S5. TEM–EDS mapping images and SAED pattern analysis

(a) Scheme illustration of precursor transient partially liquid like phase (I) and forming new crystal (II) upon IPL irradiation. (b) TEM images and EDS mapping showing particle composition and structure with shot number. Scale bar of 1 shot, 10 shot is 500 nm and scale bar of 20 shot is 200 nm. (c) Quantitative particle size analysis of IPL_WH determined from SEM images. (d) Schematic illustration of progressive H₂O partial vaporization reduces particle size while increasing particle number, leading to whitlockite crystal stabilization upon repeated irradiation.

FIGURE S6. Bulk SEM image of carbon paper

SEM images of precursor mixture (DCPD + Mg(OH)₂), precursor on carbon paper without IPL, and precursor on carbon paper with IPL treatment (left to right).

FIGURE S7. Raman spectral analysis under different samples in the same IPL irradiation conditions.

(a) Normalized Raman spectral of bare carbon paper, IPL_CP (carbon paper w/o precursor), and IPL_WH (carbon paper w/ precursor). (b) Quantitative comparison of the I_D_/I_G_ ratio (%) for the three samples. IPL_WH shows the highest I_D_/I_G_ ratio, indicating increase structural disorder or defect density compared with the other samples.

FIGURE S8. Effective of H2O vapor confinement on IPL induced phase transformation

(a) Optical images of samples prepared with different glass slide gap distances (1 mm and 5 mm). Scale bar: 5 mm. (b) FTIR spectra of IPL-processed samples under 1 mm and 5 mm gap conditions, demonstrating enhanced peak resolution and a well-resolved HPO₄²⁻ absorption band at ~930 cm⁻¹ in the 1 mm condition compared to the broad, poorly resolved absorption observed in the 5 mm condition.

FIGURE S9. Phase diagram calculation of CaHPO_4_–Mg(OH)_2_–H_2_O

Calculation phase diagram of CaHPO_4_–Mg(OH)_2_–H_2_O phase using FactSage 8.3 thermodynamic calculation.[2, 3] Phase diagram was calculated using the well-available thermodynamic data for the Ca-Mg-P-O-H system except a whitlockite phase of which Gibbs energy was unknown and roughly determined in this study.

FIGURE S10. Equilibrium calculation of DCPA(CaHPO_4_) and DCPD(CaHPO_4_·2H_2_O)

(a, b) Equilibrium calculation 1 mol of DCPA and DCPD of FactSage. DCPA releases 0.5 mol H_2_O from 1 mol DCPA, while DCPD releases 2.5 mol H_2_O from 1 mol DCPD. There are 5 times more H_2_O release on the same number of moles basis.

FIGURE S11. XPS analysis before IPL treatment was performed for each precursor.

In the case of anhydrous precursor, (a) after IPL treatment peak shifting to the right side. While hydrate precursor (b), there are no shift about before and after IPL treatment. Peak fitting analysis (right side) shows that only hydrate precursor containing HPO_4_^2-^ bonding.

Figure S12. In vitro cytocompatibility evaluation of IPL-synthesized whitlockite

(a) Cell viability of MC3T3-E1 cells assessed by CCK-8 assay with 100% eluate prepared at 1-, 2-, and 3-day extraction time points (n = 4). (b) Representative Live/Dead fluorescence staining images of MC3T3-E1 cells and portable SEM images of cell adhesion and spreading morphology on the carbon paper surface after 24 h of culture.

FIGURE S13. Thermal uniformity of IPL system

(a) Photograph of the IPL system showing the actual equipment scale. (b) Average surface temperature of bare carbon paper substrate measured by IR camera under IPL irradiation (325 V 20 ms). (c) Position-dependent temperature measurements at three spatially distinct regions (left, middle, and right) of the carbon paper substrate (n = 3), confirming spatial thermal uniformity of 1,291 ± 7 °C across the sample are

Table 1. Comparison of synthesis methods, conditions, and characteristics of whitlockite reported in the literature and current work

|  | **Synthesis method** | **Synthesis time** | **Particle size** | **Phase**  **purity** | **Crystallinity** | **Reproducibility** | **Citation** |
| --- | --- | --- | --- | --- | --- | --- | --- |
| **1** | Wet chemical precipitation | 24 h | 50 nm | Single phase | High | N/R | ACS nano 2014 Vol. 8  [4] |
| **2** | Wet chemical precipitation | 18 h | 100 nm | Single phase | Low | N/R | Nanomaterials 2020 Vol. 10  [5] |
| **3** | Wet chemical precipitation | 12 h | 25 nm | Mixed phase | Middle | N/R | Journal of Colloid and Interface Science 2020 Volume 569  [6] |
| **4** | Wet chemical precipitation | 1-21 day | 200 nm | Mixed phase | Middle | N/R | J. Am. Ceram. Soc., 2016 99 [4]  [7] |
| **5** | Microwave | 10 min,  dried 12 h | 300 nm | Single phase | High | N/R | Desalin. Water Treat. 2019 162  [8] |
| **6** | Hydrothermal | 4.5 h | 45 nm | Single phase | High | N/R | Colloids Surf. B Biointerfaces  2024 Volume 239  [9] |
| **7** | Wet chemical precipitation | 3 h,  annealing 5 h | 50 nm | Single phase | High | N/R | CrystEngComm, 2023, 25, 4370  [10] |
| **8** | Wet chemical precipitation | 24 h | 2-30 nm | Single phase | Middle | N/R | Ceram. Int. 2023 Volume 49  [11] |
| **9** | Microwave | 1 h,  dried 24h | 0.5 – 1 $\mu$m | Single phase | Middle | N/R | RSC Adv., 2016, 6, 33393-33402  [12] |
| **10** | Wet chemical precipitation | 24 h | 30-230 nm | Single phase | Middle | N/R | Ceram. Int. 2022 Volume 48  [13] |
| **11** | Wet chemical precipitation | 24 h | 50 nm | Single phase | High | N/R | Mater. Chem. B. 2015, 3  [14] |
| **12** | Hydrothermal | 3 h,  dried 12 h | 100 nm | Single phase | High | N/R | ACS Biomater. Sci. Eng. 2021, 7  [15] |
| **13** | Solid state | 6 h | 45 nm | Mixed phase | High | Confirmed  (Bulk test) | Ceram. Int. 2022 Volume 48  [16] |
| **14** | Solid state | 5 h | 1 $\mu$m | Mixed phase | High | Confirmed  (n $\geq3$) | Open Ceramics 2024 Volume 19  [17] |
| **15** | IPL synthesis | 20 ms $\leq$ | ~200 nm | Single phase | N/R | Confirmed  (n $\geq3$) | Current work |

**References**

1.	S. J. Grabowski, “Red- and Blue-Shifted Hydrogen Bonds: the Bent Rule from Quantum Theory of Atoms in Molecules Perspective,” *The Journal of Physical Chemistry A 115*	no.45 (2011): 12789-12799, <https://doi.org/10.1021/jp203908n>

2.	C. W. Bale, E. Bélisle, P. Chartrand, S. A. Decterov, G. Eriksson, A. E. Gheribi, K. Hack, I. H. Jung, Y. B. Kang, J. Melançon, A. D. Pelton, S. Petersen, C. Robelin, J. Sangster, P. Spencer, M. A. Van Ende, “Reprint of: FactSage thermochemical software and databases, 2010–2016,” *Calphad 55*	(2016): 1-19, <https://doi.org/10.1016/j.calphad.2016.07.004>

3.	I.-H. Jung, M.-A. Van Ende, “Computational Thermodynamic Calculations: FactSage from CALPHAD Thermodynamic Database to Virtual Process Simulation,” *Metallurgical and Materials Transactions B 51*	no.5 (2020): 1851-1874, <https://doi.org/10.1007/s11663-020-01908-7>

4.	H. L. Jang, K. Jin, J. Lee, Y. Kim, S. H. Nahm, K. S. Hong, K. T. Nam, “Revisiting whitlockite, the second most abundant biomineral in bone: nanocrystal synthesis in physiologically relevant conditions and biocompatibility evaluation,” *ACS nano 8*	no.1 (2014): 634-641, <https://doi.org/10.1021/nn405246h>

5.	S. Batool, U. Liaqat, Z. Hussain, M. Sohail, “Synthesis, characterization and process optimization of bone whitlockite,” *Nanomaterials 10*	no.9 (2020): 1856, <https://doi.org/10.3390/nano10091856>

6.	C. Wang, K.-J. Jeong, H. J. Park, M. Lee, S.-C. Ryu, D. Y. Hwang, K. H. Nam, I. H. Han, J. Lee, “Synthesis and formation mechanism of bone mineral, whitlockite nanocrystals in tri-solvent system,” *Journal of Colloid and Interface Science 569*	(2020): 1-11, <https://doi.org/10.1016/j.jcis.2020.02.072>

7.	A. C. Tas, “Transformation of brushite (CaHPO4· 2H2O) to whitlockite (Ca9Mg (HPO4)(PO4) 6) or other CaPs in physiologically relevant solutions,” *Journal of the American Ceramic Society 99*	no.4 (2016): 1200-1206, <https://doi.org/10.1111/jace.14069Digital>

8.	C. Lin, Y. Wang, Y. Zhou, Y. Zeng, “A rapid way to synthesize magnesium whitlockite microspheres for high efficiency removing heavy metals,” *Desalination and Water Treatment 162*	(2019): 220-227, <https://doi.org/10.5004/dwt.2019.24290>

9.	L. V. Maximiano, L. B. Correa, N. C. Gomes-da-Silva, L. S. da Costa, M. G. P. Da Silva, A. V. Chaves, M. L. Franco, P. B. A. Fechine, A. S. de Menezes, R. Santos-Oliveira, “Magnesium whitlockite nanoparticles: Hydrothermal synthesis, anti-inflammatory and anti-cancer potential,” *Colloids and Surfaces B: Biointerfaces 239*	(2024): 113931, <https://doi.org/10.1016/j.colsurfb.2024.113931>

10.	A. Kizalaite, V. Klimavicius, V. Balevicius, G. Niaura, A. N. Salak, J.-C. Yang, S. H. Cho, T. Goto, T. Sekino, A. Zarkov, “Dissolution–precipitation synthesis and thermal stability of magnesium whitlockite,” *CrystEngComm 25*	no.30 (2023): 4370-4379, <https://doi.org/10.1039/D3CE00602F>

11.	A. Afonina, A. Dubauskas, V. Klimavicius, A. Zarkov, A. Kareiva, I. Grigoraviciute, “Phase transformations during the dissolution-precipitation synthesis of magnesium whitlockite nanopowders from gypsum,” *Ceramics International 49*	no.23 (2023): 38157-38164, <https://doi.org/10.1016/j.ceramint.2023.09.146>

12.	C. Qi, F. Chen, J. Wu, Y.-J. Zhu, C.-N. Hao, J.-L. Duan, “Magnesium whitlockite hollow microspheres: a comparison of microwave-hydrothermal and conventional hydrothermal syntheses using fructose 1, 6-bisphosphate, and application in protein adsorption,” *RSC advances 6*	no.40 (2016): 33393-33402, <https://doi.org/10.1039/C6RA00775A>

13.	A. Afonina, A. Kizalaite, A. Zarkov, A. Drabavicius, T. Goto, T. Sekino, A. Kareiva, I. Grigoraviciute-Puroniene, “Synthesis of whitlockite nanopowders with different magnesium content,” *Ceramics International 48*	no.21 (2022): 32125-32130, <https://doi.org/10.1016/j.ceramint.2022.07.152>

14.	H. L. Jang, H. K. Lee, K. Jin, H.-Y. Ahn, H.-E. Lee, K. T. Nam, “Phase transformation from hydroxyapatite to the secondary bone mineral, whitlockite,” *Journal of Materials Chemistry B 3*	no.7 (2015): 1342-1349, <https://doi.org/10.1039/C4TB01793E>

15.	A. Kizalaite, I. Grigoraviciute-Puroniene, D. R. C. Asuigui, S. L. Stoll, S. H. Cho, T. Sekino, A. Kareiva, A. Zarkov, “Dissolution–precipitation synthesis and characterization of zinc whitlockite with variable metal content,” *ACS Biomaterials Science & Engineering 7*	no.8 (2021): 3586-3593, <https://doi.org/10.1021/acsbiomaterials.1c00335>

16.	S. Batool, Z. Hussain, U. Liaqat, M. Sohail, “Solid-state synthesis and process optimization of bone whitlockite,” *Ceramics International 48*	no.10 (2022): 13850-13854, <https://doi.org/10.1016/j.ceramint.2022.01.267>

17.	M. Bohner, F. Bigolin, I. Bohner, T. Imwinkelried, Y. Maazouz, P. Michel, C. Stähli, Y. Viecelli, N. Döbelin, “The reactivity of α-tricalcium phosphate powders is affected by minute amounts of β-calcium pyrophosphate and by the synthesis temperature,” *Open Ceramics 19*	(2024): 100647, <https://doi.org/10.1016/j.oceram.2024.100647>
